# Comparative morphology and trophic ecology in a population of the polymorphic lizard *Sceloporus minor* (Squamata: Phrynosomatidae) from central Mexico

**DOI:** 10.7717/peerj.8099

**Published:** 2019-11-22

**Authors:** Aaron García-Rosales, Aurelio Ramírez-Bautista, Barry P. Stephenson

**Affiliations:** 1 Laboratorio de Ecología de Poblaciones, Centro de Investigación Biológica, Instituto de Ciencias Básicas e Ingeniería, Universidad Autónoma del Estado de Hidalgo, Pachuca, Hidalgo, México; 2 Department of Biology, Mercer University, Macon, GA, United States of America

**Keywords:** Polymorphism, Diet, Population, Lizards, Morphometry

## Abstract

Polymorphism among individuals of the same population has generally been linked to alternative reproductive tactics, where different morphs can exhibit differences in their morphological, ecological, and behavioral attributes. These differences may result in a divergence in diet between morphs due to differential exploitation of habitat, morphological differences that influence prey selection, or differential energy expenditure that results in different nutritional needs. The present study analyzes the morphology (morphometry and body mass) and diet of red and yellow male morphs in a population (El Enzuelado) of the lizard *Sceloporus minor* from central Mexico. No differences between morphs were found for any of the morphometric variables analyzed (snout-vent length, tail length, jaw length, jaw width, head length, head width, head height, tibia length, femur length, forearm length and ventral patch length). In both morphs, allometric growth was observed in all body features analyzed, as well as in morphometric features of the head across seasons. Analysis of stomach contents showed that the diet of red males was composed of 12 categories of prey, while that of yellow males was composed of 10 categories; those categories of diet not shared between morphs (e.g., Isoptera, Psocoptera) were consumed by their respective morph in very low proportions. Categories of diet with the highest values of food importance for both groups were Coleoptera, Orthoptera, and leaves; a similar pattern was seen across seasons. This, in turn, is reflected in low niche breadth values for each morph and a very high niche overlap. There were no significant differences between morphs overall, or between morphs per season, in the weight and volume of stomach contents or in the number of prey items found in stomachs; however, differences in these variables across all males (independent of morph) were recorded between seasons. Likewise, no significant correlations were found between body size (snout-vent length) and the volume of stomach contents for either morph or between lizard mandibular dimensions and the volume of stomach contents for red morph males. For the yellow morph, prey volume unexpectedly decreased significantly with jaw size rather than increasing as expected. Overall, this study adds new information about the morphology and feeding of males in this species, and suggests that in this population, color morphs lack the morphological and ecological differences found in some other species of polymorphic lizard.

## Introduction

The presence of two or more color morphs (polymorphism) among individuals of the same sex and population is a topic that has received increasing attention from researchers in recent years (Sinervo et al., 2000; Zamudio & Sinervo, 2000; Hamilton & Sullivan, 2005; Bastiaans et al., 2013; Olsson, Stuart-Fox & Ballen, 2013; Lattanzio & Miles, 2016; Scali et al., 2016; McDiarmid et al., 2017; Paterson & Blouin-Demers, 2018). Many of these studies attempt to identify the processes that lead to the evolution and maintenance of links between alternative color morphs and morph-specific behavioral strategies (Sinervo & Lively, 1996; Sinervo et al., 2000; Zamudio & Sinervo, 2000). Such intrasexual polymorphism is usually linked to alternative reproductive tactics (ARTs; Gross, 1996; Taborsky, Oliveira & Brockmann, 2008), which represent alternative pathways to reproductive success among members of a single sex, and the expression of multiple interrelated phenotypes working in concert to maximize fitness (Taborsky, Oliveira & Brockmann, 2008). Maintaining the polymorphism requires that each morph achieve equal fitness over a long period of time (Gross, 1996; Taborsky, Oliveira & Brockmann, 2008). This balance can be achieved through two processes, niche partitioning (Skúlason & Smith, 1995; Lattanzio & Miles, 2016; Scali et al., 2016; Paterson & Blouin-Demers, 2018), and frequency-dependent selection (Sinervo & Lively, 1996; Pryke et al., 2007).

The niche partitioning hypothesis proposes that individuals from different morphs exploit different resources of the environment (e.g., space, shelter, food) to avoid strong competition for the same resource (Skúlason & Smith, 1995; Lattanzio & Miles, 2016; Scali et al., 2016; Paterson & Blouin-Demers, 2018). For example, morphs of *Podarcis muralis* Laurenti, 1768 and *Urosaurus ornatus* Baird & Girard, 1852, have shown preferences for certain types of prey, only partially shared with other morphs of the same species (Lattanzio & Miles, 2016; Scali et al., 2016; Paterson & Blouin-Demers, 2018); in addition, differences in the spatial distribution of morphs have been recorded in *U. ornatus* (Paterson & Blouin-Demers, 2018). Negative frequency-dependent selection can also maintain a polymorphism by conferring advantages of survival and / or reproduction to rare morphs, as the fitness of a given phenotype depends on the frequencies of the other phenotypes with which it is competing (Sinervo & Lively, 1996; Pryke et al., 2007). For example, frequency-dependent selection maintains the polymorphism in the lizard *Uta stansburiana* Baird & Girard, 1852. In this species, the frequency of each morph changes across years in a cyclic manner, a consequence of the different behavioral strategies exhibited by the different morphs (Sinervo & Lively, 1996).

In lizards, differences between morphs have generally been linked to differences in spatial dispersion within the landscape, body size, aggression, territory and/or home range size, and the quality of the habitats used (Thompson & Moore, 1992; Sinervo & Lively, 1996; Bustos-Zagal et al., 2014). These differences could indicate that different morphs are capable of exploiting different components of a resource gradient, and consequently that trophic niches could also diverge (Lattanzio & Miles, 2016). Consequently, the expression of ARTs in different color morphs could favor the segregation of such morphs in the environment, in turn allowing each morph to exploit different types of prey linked to their respective habitats (Lattanzio & Miles, 2016). This divergence of trophic niche would therefore favor the maintenance of polymorphism in these populations (niche partitioning hypothesis; Lattanzio & Miles, 2016; Scali et al., 2016).

Likewise, in polymorphic species of lizards that exhibit ARTs, it is common to observe that one morph is territorial, and the other is not (Thompson & Moore, 1992; Sinervo & Lively, 1996). Given possible differences in energy intake and expenditure between territorial and non-territorial individuals, and that prey may differ in the quantity and quality of their nutrients, morphs might also be expected to differ in their diet (Leyte-Manrique & Ramírez-Bautista, 2010; Cruz-Elizalde et al., 2014). In addition, morph-specific variation in coloration may be associated with other morphological traits, such as body size (snout-vent length; SVL) and/or head size (Hover, 1985; Sinervo & Lively, 1996; Moore, Hews & Knapp, 1998; Sinervo et al., 2000; Bustos-Zagal et al., 2014). Some of these morphological features that distinguish each morph could also act as a constraint on the type of prey that each can consume, further driving a divergence in diet. For example, at the population level, prey size and volume are in some cases correlated with dimensions of the head, which may in turn promote variation in diet between sexes and age classes (Ballinger, 1977; Cooper, Lemos-Espinal & Smith, 1998; Herrel et al., 2006). In addition, individuals of small size tend to consume small and soft prey, in contrast to the larger and harder prey consumed by large individuals of the same species (Gadsden et al., 2011; Ngo et al., 2015).

Consequently, in species that exhibit polymorphism linked to ARTs, morph-specific divergence in diet could occur through at least three mechanisms: (1) differential exploitation of habitats, with each morph eating different prey found in these different habitats; (2) differential energy expenditure and hence different nutritional needs between morphs that influence that type of prey consumed; and (3) morphological differences between morphs that in turn influence the type of prey consumed. To date, few studies have analyzed both morphology (Thompson & Moore, 1992; Sinervo & Lively, 1996; Bustos-Zagal et al., 2014) and diet (Lattanzio & Miles, 2016; Scali et al., 2016) in polymorphic lizard species; such information is necessary to identify the specific mechanisms responsible for maintaining polymorphisms in a population (Lattanzio & Miles, 2016; Scali et al., 2016).

The genus *Sceloporus* Wiegmann, 1828 (Phrynosomatidae) is represented by 106 species (Uetz, Freed & Hošek, 2018) distributed from Canada to Central America (Smith, 1939). The species *S. minor* Cope, 1885 is endemic to Mexico, occurring in the central and northern regions of the country in the states of San Luis Potosí, Querétaro, Guanajuato, Zacatecas, Nuevo León, and Hidalgo (Wiens, Reeder & Nieto-Montes de Oca, 1999). *Sceloporus minor* is a species that has been relatively well studied in certain aspects of its biology, such as systematics, reproduction, and morphology (Wiens, Reeder & Nieto-Montes de Oca, 1999; Ramírez-Bautista et al., 2008; Stephenson, 2010; Stephenson & Ramírez-Bautista, 2012; Ramírez-Bautista et al., 2014; García-Rosales et al., 2017). Morphological studies conducted within and between populations of this species have shown marked within- and between-sex phenotypic variation in several traits (Stephenson, 2010; Stephenson & Ramírez-Bautista, 2012; García-Rosales et al., 2017). Specifically, intersexual variation has been reported in the coloration of the dorsum, throat, and ventral abdominal patches, as well as SVL, in that males were found to be more colorful and larger than females (see García-Rosales et al., 2017). Intrasexual variation has been observed in the dorsal coloration of males (which can be red, brown, yellow, or blue: Wiens, Reeder & Nieto-Montes de Oca, 1999; Stephenson & Ramírez-Bautista, 2012; García-Rosales et al., 2017), as well in SVL, head size, and physiology (Stephenson, 2010; but see García-Rosales et al., 2017). Despite these advances, there are many unknown details regarding intrasexual variation in other characteristics, including details of morphology, diet, and behavior. Such studies are relevant because they provide new information about polymorphic lizard species, in turn allowing us to more fully understand their ecology.

In this study, we analyzed aspects of morphology and diet in red and yellow morphs of adult males from a population of *S. minor* in central Mexico. Our specific objectives were to: (1) compare morphometric characteristics between morphs, (2) analyze the overall diet composition of each morph, (3) analyze the diet composition of each morph across three seasons of the year (summer, fall, and spring), (4) determine the dietary niche breadth and overlap between morphs, and (5) analyze the relationship between body size and mandibular dimensions with the volume of stomach content and prey sizes (respectively) for each male morph. If the niche partitioning hypothesis helps maintain the presence of polymorphism in this population, morphs should show differences in at least some of their morphological or trophic ecology attributes that would allow them to differentially exploit resources in the environment, thus helping reduce resource competition between morphs. Alternatively, if these morphs do not differ in any of their trophic or morphological features, this would suggest that polymorphism in this population is being maintained by frequency-dependent natural selection.

## Material and Methods

### Study area

Fieldwork was conducted at a site approximately five hectares in size near the community of El Enzuelado (20°35′N, 98°37′W) of the municipality of San Agustín Metzquititlán, Hidalgo, Mexico. El Enzuelado is located at an elevation of 1,955 m, and the dominant vegetation type is xerophilous scrub (Rzedowski, 1978). The mean annual temperature is 17.5 °C and mean annual precipitation is 496.7 mm (Pavón & Meza-Sánchez, 2009).

### Sampling

**Figure 1:**
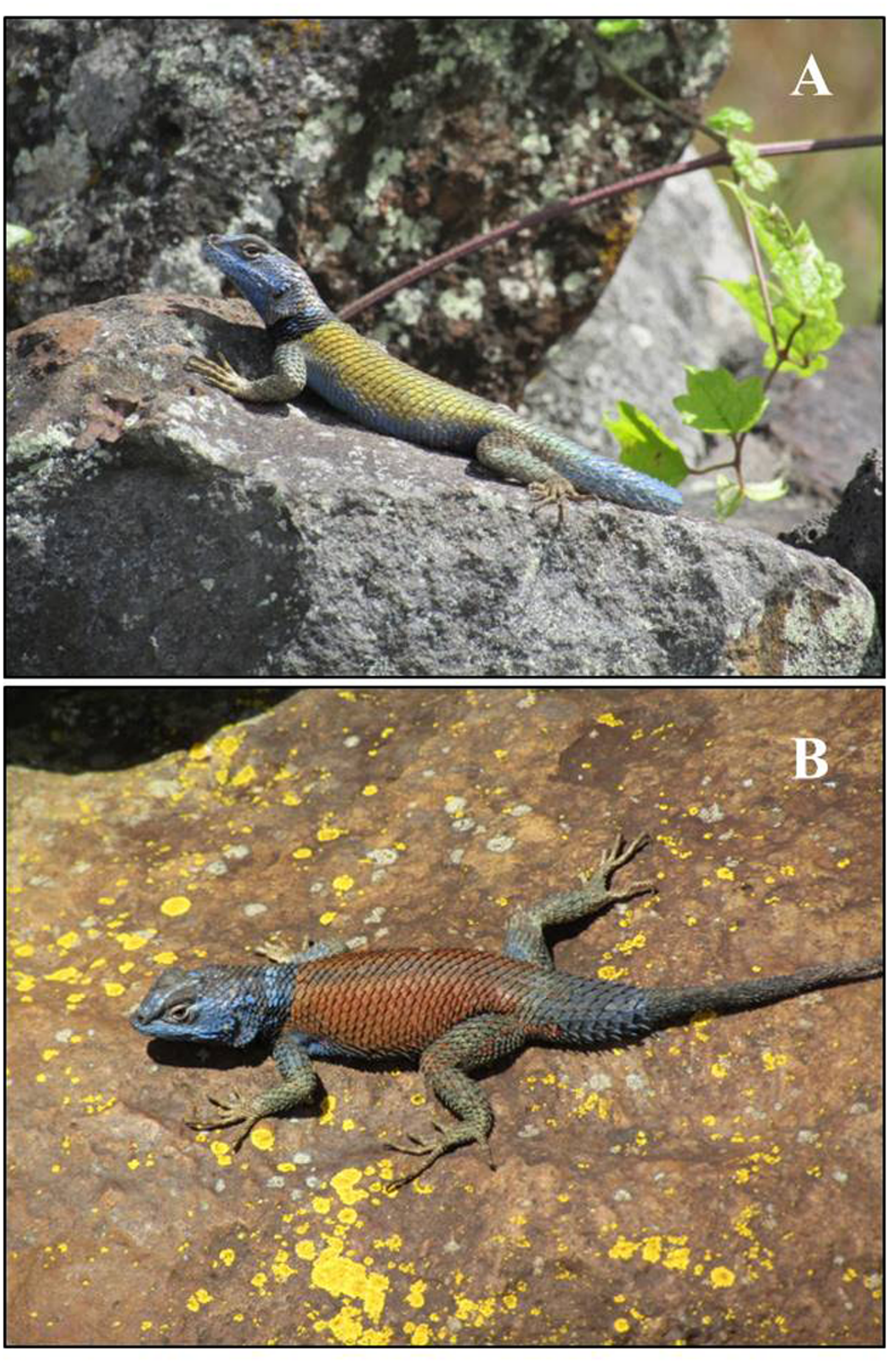
Representative examples of variation in dorsal color pattern in male *S. minor* from El Enzuelado, Hidalgo, México. A-yellow, B-red. photographs taken by Aaron García-Rosales.

Surveys for adult male lizards were conducted from June 2017 to March 2018. Snout-vent length of adults corresponded to those of Ramírez-Bautista et al. (2014). In total, we measured 99 adult males (58 red males and 41 yellow males; Fig. 1). Specimens were collected in June (summer; 11 red and nine yellow males) and November (fall; 41 red and 25 yellow males) 2017, and in March (spring; six red and seven yellow males) 2018. All searches were conducted between 10:00-17:00 h, and were limited to boulders and rock piles, the microhabitats in which adult males of this species are most commonly observed at this site (A García-Rosales, pers. obs., 2017); once lizards were found, they were captured directly by hand. Sex identification was made on the basis of the (sexually dimorphic) color patterns of each lizard, as well as examination of the enlarged post-anal scales present only in males.

This study was conducted according to the ethics and regulations for animal research of the Universidad Autónoma del Estado de Hidalgo, the AVMA Guidelines on Euthanasia (AVMA 2013), and the policies for handling of animal specimens described in the NORMA Oficial Mexicana NOM-033-SAG/ZOO-2014. All animal use was approved under collecting permit SGPA/DGVS/06183/17 issued by the Secretaría del Medio Ambiente y Recursos Naturales (SEMARNAT) of the Government of Mexico.

### Morphometric analysis

We collected data on 11 morphometric variables from each lizard in the field. Distance measures [snout-vent length (SVL; distance from the tip of the rostral scale to the cloaca), tail length (TLL; distance from the vent to the tip of the tail), jaw length (JL; distance from the tip of the rostral scale to the point of maximum width of the left side of the mandible), jaw width (JW; the maximum distance between the left and right sides of the mandible), head length (HL; distance from the anterior tip of the rostral scale to the posterior margin of the left ear), head width (HW; maximum width of the head, measured as the distance between the posterior margin of the left and right ears), head height (HH; the maximum distance between the dorsal and ventral sides of the head), tibia length (TL; distance from the knee to the pad of the foot), femur length (FL; distance from the angle of the groin to the knee), forearm length (FOL; distance from the elbow to the pad of the foot), and ventral patch length (VPL; the maximum longitudinal distance of the dark edge)] were collected with the aid of a Mitutoyo digital caliper (±0.01 mm). Body mass was measured using a Pesola spring scale (±0.01 g). In addition, we recorded the dorsal color of each individual based on a color catalogue for field work (Köhler, 2012) in the context of previously described color morph variation in males from El Enzuelado (see García-Rosales et al., 2017; Fig. 1). Following morphometric data collection, most individuals (54 of 99 males) were permanently marked by toe clipping, then released at their site of capture to avoid measuring the same lizard on multiple occasions (García-Rosales & Martínez-Coronel, 2016).

To evaluate morphometric differences between color morphs, we used a discriminant function analysis that included body mass, SVL, TLL, JL, JW, HL, HW, HH, TL, FL, FOL, and VPL for both groups (i.e., red and yellow morphs). Differences between groups were estimated, and we identified those variables that provided the greatest variation between groups. Before analysis, all means were transformed into Z scores using the formula $Z= \left( \frac{X-\mu }{\sigma } \right) $, standardizing the data. To characterize morph allometry, we performed multiple regressions for each morph using the 10 morphometric variables described above and body mass as dependent variables and SVL as the independent variable. In addition to this overall analysis, we also assessed allometric patterns across individual seasons (spring, summer, and fall); for this latter analysis, a subset of morphometric head measurements (HL, HW, HH, JL, and JW) were used as dependent variables in each season, with SVL as the independent variable, as before. Tests were considered significant if *P* ≤ 0.05. Statistical tests for this analysis were performed using Statistica 7.0 (StatSoft, Inc., Tulsa, OK, USA).

### Dietary analysis

The remaining 45 males (24 red and 21 yellow morphs) that were not released were used in an analysis of stomach contents, as well as to collect other data not presented here. These lizards were collected in the month of June (summer; 11 red and nine yellow males) and November (fall; seven red and five yellow males) of 2017, and in March (spring; six red and seven yellow males) of 2018. All lizards used in this analysis were collected between 13:00 and 17:00 h, in order to minimize the likelihood of finding individuals with an empty stomach, and were humanely euthanized in the laboratory via intracoelomic injection of sodium pentobarbital. Specimens were then fixed in 10% formalin and preserved in 70% ethanol (Ramírez-Bautista et al., 2008). All of these specimens were deposited at the collection of Amphibians and Reptiles of the Centro de Investigaciones Biológicas, Universidad Autónoma del Estado de Hidalgo.

Before fixing in formalin, the stomach of each collected lizard was removed and weighed using an Adam analytical balance (±0.0001 g). Stomach contents were removed and placed in Petri dishes, and the length, width, and height of each prey item were recorded using Mitutoyo digital calipers (±0.01 mm; (Leyte-Manrique & Ramírez-Bautista, 2010). The volume (mm^3^) of all stomach contents (the total volume of all prey items in the stomach), volume of each category of prey found in a stomach (total volume of all grouped prey of the same order or category found in a single stomach), and the volume of each individual intact prey item found in each lizard stomach were obtained using the formula of an ellipsoid (Duré, Kehr & Schefer, 2009): $V= \frac{4}{3} \pi \ast \left[ \left( \frac{length}{2} \right) \ast { \left( \frac{width}{2} \right) }^{2} \right] $. We used dichotomous keys to identify most types of prey to the taxonomic level of order (Triplehorn & Johnson, 2005), with the exception of Hymenoptera, which were further classified as formicids (ants) and non-formicids (bees and wasps). In addition, holometabolous prey items were identified as being either larvae or adults, and each was treated as an independent category (Aldape-López, Lazcano-Hernández & Martínez-Coronel, 2009; Gadsden et al., 2011). Plant matter was classified as leaves, flowers, or fruits (Hernández-Salinas, Ramírez-Bautista & Cruz-Elizalde, 2016).

We used the relative importance index (*I*) to determine the value of each category of consumed prey to each morph. This index includes three parameters for consumed prey (frequency, number, and volume), and is calculated as $I= \frac{\text{%}F+\text{%}N+\text{%}V}{3} $, where %*F* is the percentage of occurrence, %*N* is the numerical percentage, and %*V* is the volumetric percentage (Biavati, Wiederhecker & Colli, 2004; Ngo et al., 2014). The relative importance index was calculated for each morph overall, and for each morph on a per-season basis (summer, fall, and winter). We calculated niche breadth using Levin’s standardized niche index (Hurlbert, 1978), with the formula ${B}_{A}= \frac{ \left( \frac{1}{\sum p{i}^{2}} \right) -1}{n-1} $, where: *pi* is the proportion (number of individuals) in each prey category with respect to the total number of prey found in each group (color morph), and *n* is the number of each prey category found in the diet of individuals of each morph. This index is calculated by groups (e.g., morphs, season of the year) and ranges from zero to one, where zero indicates a specialist diet and one indicates a general diet (Hurlbert, 1978). Diet overlap was assessed using Pianka’s O_jk_ index (Pianka, 1973), with the formula ${O}_{jk}= \frac{{\mathop{\sum }\nolimits }_{i=1}^{n}{p}_{ij}{p}_{ik}}{\sqrt{{\mathop{\sum }\nolimits }_{i=1}^{n}{p}_{ij}^{2}}{\mathop{\sum }\nolimits }_{i=1}^{n}{p}_{ik}^{2}} $, where *P*_*ij*_ and *P*_*ik*_ are the proportions of resource *i* used by groups (i.e., morphs) *j* and *k*, respectively. This index is calculated by pairs of groups (e.g., seasons of the year, morphs) and ranges from zero to one, where zero indicates that these groups consume different prey or use different resources (i.e., there is no overlap), and one indicates that these groups consume the same categories of prey or use the same resources (maximum niche overlap; Pianka, 1973). Niche breadth was calculated for each morph separately and for all individuals, while niche overlap was calculated only between morphs. Both indices were calculated using the Ecological Methodology software, version 6.1.1 (Krebs, 1999).

We evaluated differences between morphs in the volume and weight of stomach contents, as well as the number of prey consumed. All data were initially log_10_ transformed to meet normality assumptions for parametric tests. When normality was met, we used *t*-tests for subsequent comparisons; for data sets that were not normalized following transformation, we used Mann–Whitney *U* tests with untransformed data instead (Zar, 1999). These same variables (volume and weight of stomach contents, and number of prey consumed) were also evaluated for each morph through three seasons of the year (summer, fall, and spring) using a MANOVA. For this analysis, all response variables were transformed to Z scores (see formula in *Morphometric Analysis*). In addition, we assessed the relationships between body size (SVL) and the volume of stomach contents and total number of prey, as well as relationships between two variables of head size (JL and JW) and the total volume of prey in the stomach. As before, data were initially log_10_ transformed in an attempt to meet normality conditions for parametric tests. When these conditions were met, we used Pearson (*r*) correlation tests; when data failed to normalize, we used Spearman correlations (*r*_s_) on the untransformed data instead (Zar, 1999). Tests were considered significant if *P* ≤ 0.05. Statistical analyses were performed using Statistica 7.0 (StatSoft, Inc., Tulsa, OK, USA). Means are presented as $\bar {X}$ ± standard deviation.

## Results

The discriminant function analysis showed that red and yellow morph males have a similar morphology overall, since no differences between morphs were observed in any morphometric variable (Wilks’ lambda = 0.97, *F*_(1,97)_ = 2.77, *P* = 0.99; Table 1). Multiple regression analysis did find a significant positive relationship between SVL and most morphometric variables analyzed for each morph (red: *R*^2^ = 0.07, *F*_11,46_ = 53.73, *P* < 0.01; yellow: *R*^2^ = 0.08, *F*_11,29_ = 43.68, *P* < 0.01; Table 2), with the exception of TLL in red males, and TLL and FOL in yellow males (Table 2). Multiple regressions by season on the head measurements only showed that for the red morph, there was a positive and significant relationship in the fall (*R*^2^ = 0.78, *F*_5,35_ = 75.4; *P* < 0.01), but not the summer (*R*^2^ = 0.35, *F*_5,5_ = 1.35, *P* = 0.37), and an undetermined relationship in the spring (*R*^2^ = 0.79, F and P values were not calculated due to insufficient data). Similarly, multiple regressions revealed a significant positive relationship between head measurements and SVL for the yellow morph in the fall (*R*^2^ = 0.82, *F*_5,19_ = 41.24, *P* < 0.01); these relationships were positive but nonsignificant among males from the spring (*R*^2^ = 0.74, *F*_5,1_ = 5.39, *P* = 0.31) and summer (*R*^2^ = 0.25, *F*_5,3_ = 4.1, *P* = 0.13). For both morphs, simple regressions revealed positive relationships between SVL and the individual head variables analyzed in each of these three seasons, but not all were significant (Table 3).

**Table 1:** Descriptive statistics of morphometric traits and summary data from discriminant function analysis of red and yellow male morphs of *Sceloporus minor* from El Enzuelado. Morphometric measurements reported as mean (in mm) ± standard deviation.

	Red (*n* = 58)	Yellow (*n* = 41)	Wilks’ lambda	F	*p*-value
Body mass (g)	15.3 ± 4.1	15.2 ± 4.7	0.972	0.047	0.82
Snout-vent length	73.0 ± 7.3	72.9 ± 8.0	0.972	0.044	0.83
Tail length	100.8 ± 22.6	92.9 ± 24.3	1.000	2.766	0.10
Head length	17.4 ± 1.9	17.4 ± 2.4	0.972	0.037	0.84
Head width	15.9 ± 1.8	15.7 ± 2.1	0.971	0.083	0.77
Head height	9.7 ± 1.3	9.8 ± 1.4	0.970	0.212	0.64
Jaw length	11.9 ± 1.1	11.9 ± 0.9	0.972	0.025	0.87
Jaw width	13.8 ± 1.3	13.7 ± 1.4	0.972	0.002	0.96
Femur length	15.7 ± 1.6	15.8 ± 1.9	0.969	0.306	0.58
Tibia length	12.8 ± 1.2	13.0 ± 1.2	0.965	0.677	0.41
Forearm length	10.8 ± 1.2	13.4 ± 16.3	0.965	0.772	0.38
Ventral patch length	35.6 ± 7.2	34.4 ± 4.5	0.969	0.323	0.57

**Table 2:** Summary of simple regression results from multiple regression analysis of all males of *Sceloporus minor*. A total of 10 morphometric variables and body mass were used as dependent variables, with SVL as the independent variable.

	Red		Yellow	
	R	*p*-value	R	*p*-value
Body mass	0.94	<0.01	0.94	<0.01
Tail length	0.09	0.50	0.25	0.11
Head length	0.90	<0.01	0.71	<0.01
Head width	0.89	<0.01	0.89	<0.01
Head height	0.74	<0.01	0.87	<0.01
Jaw length	0.83	<0.01	0.81	<0.01
Jaw width	0.82	<0.01	0.90	<0.01
Femur length	0.81	<0.01	0.92	<0.01
Tibia length	0.50	<0.01	0.84	<0.01
Forearm length	0.80	<0.01	0.11	0.47
Ventral patch length	0.56	<0.01	0.59	<0.01

**Table 3:** Summary of simple regression results from multiple regression analysis carried out by season of the year of *Sceloporus minor*. A subset of five head measurements were used as dependent variables in each regression, with SVL as the independent variable.

	Red						Yellow					
	Spring		Summer		Fall		Spring		Summer		Fall	
	R	*p*-value	R	*p*-value	R	*p*-value	R	*p*-value	R	*p*-value	R	*p*-value
HL	0.90	0.01	0.59	0.05	0.94	<0.01	0.89	<0.01	0.24	0.53	0.90	<0.01
HW	0.93	<0.01	0.71	0.01	0.89	<0.01	0.90	<0.01	0.71	0.03	0.94	<0.01
HH	0.70	0.12	0.45	0.16	0.79	<0.01	0.87	0.01	0.87	<0.01	0.86	<0.01
JL	0.92	<0.01	0.53	0.09	0.87	<0.01	0.50	0.25	0.74	0.02	0.88	<0.01
JW	0.94	<0.01	0.54	0.08	0.85	<0.01	0.83	0.02	0.79	0.01	0.93	<0.01

**Figure 2:**
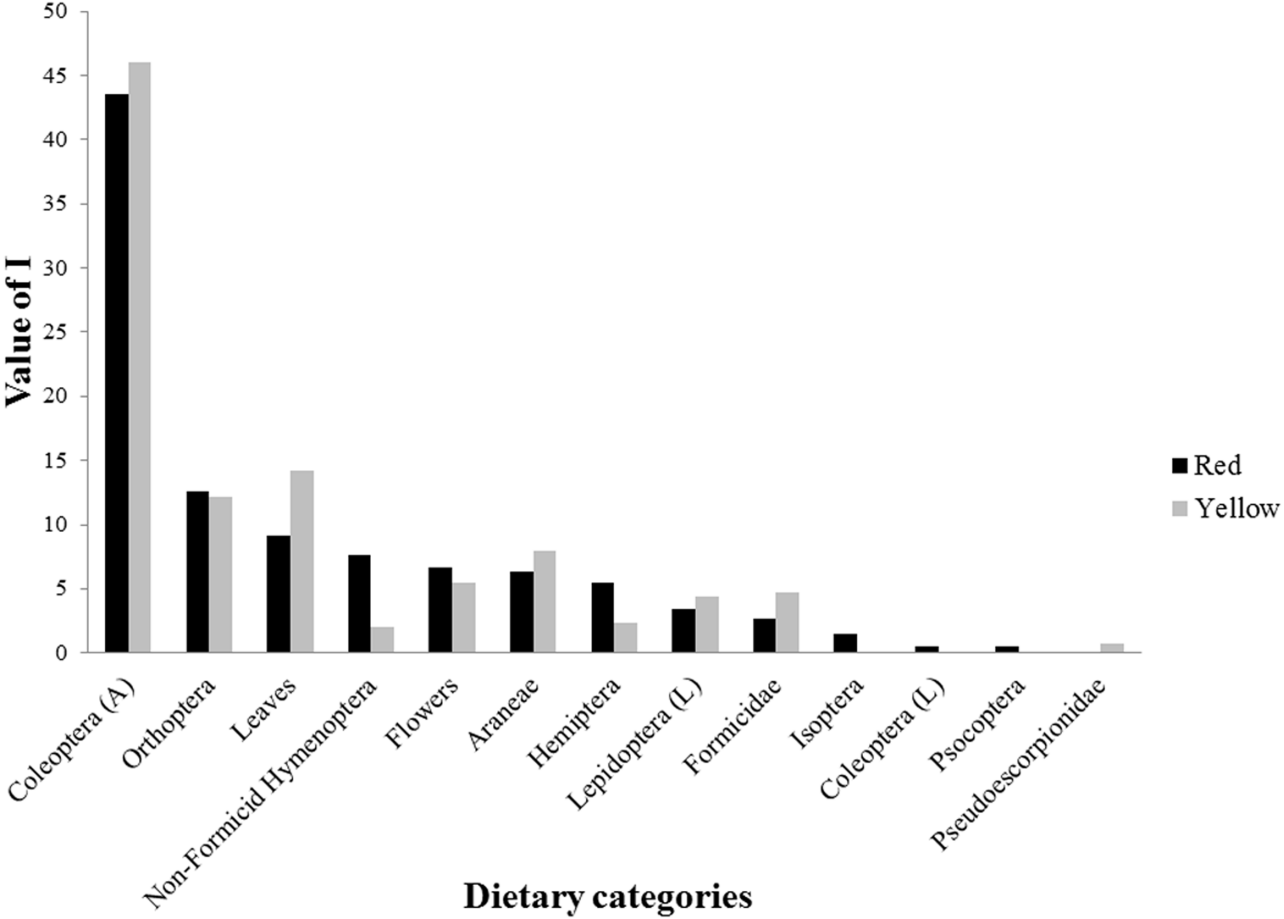
Values of food importance (I) of red and yellow male morphs of *Sceloporus minor* from El Enzuelado, Mexico. A, adults; L, larvae.

For the dietary analysis, all 45 examined individuals (24 red and 21 yellow males) were included, as no lizard was found to have an empty stomach. We identified a total of 576 prey items belonging to 13 dietary categories, including insects, arachnids, and plant matter (Fig. 2, Table 4). Of the total prey items recorded, 331 were consumed by red males and 245 by yellow males. The diet of red males was composed of 12 diet categories, while that of yellow males consisted of 10 categories. Prey categories that were not shared between morphs were Isoptera, Coleoptera larvae, Psocoptera (all of which were exclusive to the red morph), and Pseudoescorpionidae (exclusive to the yellow morph). However, all of these prey represented a small proportion of the diet of the respective morph. For both morphs, the diet categories that presented the highest values of alimentary importance were Coleoptera adults, Orthoptera, and leaves (Fig. 2, Table 4). Differences in the number of prey categories consumed by each morph were seen across different seasons (Table 5). However, the category of diet with the highest value of food importance (adult Coleoptera) was the same for both morphs across spring, summer, and fall (Table 5). Also, it was observed that plant matter (flowers and leaves) was one of the major components of the diet of these lizards, ranking higher than that of several categories of insect prey (Table 5).

**Table 4:** Diet composition of red and yellow male morphs of *Sceloporus minor* from El Enzuelado.

	Red						Yellow					
Category	F	%F	N	%N	V	%V	F	%F	N	%N	V	%V
Coleoptera (A)	21	22.34	84	40.78	59,854	67.42	19	27.14	73	51.05	34,446	59.88
Orthoptera	16	17.02	17	8.25	11,179	12.59	10	14.29	12	8.39	7,895	13.72
Leaves	15	15.96	15	7.28	3,604	4.06	13	18.57	13	9.09	8,548	14.86
Non-Formicid Hymenoptera	7	7.45	30	14.56	875	0.99	2	2.86	4	2.80	254	0.44
Flower	9	9.57	12	5.83	3,948	4.45	4	5.71	9	6.29	2,524	4.39
Araneae	9	9.57	14	6.80	2,415	2.72	9	12.86	11	7.69	1,883	3.27
Hemiptera	6	6.38	10	4.85	4,699	5.29	3	4.29	3	2.10	458	0.80
Lepidoptera (L)	5	5.32	5	2.43	2,126	2.40	5	7.14	5	3.50	1,437	2.50
Formicidae	3	3.19	10	4.85	38	0.04	4	5.71	12	8.39	77	0.13
Isoptera	1	1.06	7	3.40	33	0.04						
Coleoptera (L)	1	1.06	1	0.49	3	0.00						
Psocoptera	1	1.06	1	0.49	2	0.00						
Pseudoescorpionidae							1	1.43	1	0.70	6	0.01

**Notes.**

F frequency of occurrence %F percentage of F N number of items %N percentage of N V prey volume (mm 3) %V percentage of V A adults L larvae

**Table 5:** Value of the relative importance index of red and yellow male morphs of *Sceloporus minor* by season.

	Summer		Fall		Spring	
Category	Red	Yellow	Red	Yellow	Red	Yellow
Araneae	4.70	10.34	12.72	9.29	7.89	4.52
Coleoptera (A)	50.72	61.16	27.42	32.43	20.11	23.35
Coleoptera (L)	0.99					
Flowers	1.03		21.77	14.90	12.18	9.28
Formicidae	3.79	2.68	2.26	11.91		2.92
Hemiptera	4.62		2.69		13.61	7.31
Leaves	8.92	17.12	5.08	6.25	16.66	15.59
Non-Formicid Hymenoptera	9.64	2.68	7.19		2.67	2.62
Isoptera	2.52					
Lepidoptera (L)					19.47	13.82
Orthoptera	12.07	6.03	20.87	22.12	7.41	20.60
Pseudoescorpionidae				3.10		
Psocoptera	0.99					

**Notes.**

A adults L larvae

For all males combined, the niche breadth value was low (B_A_ = 0.263), though red males showed higher values of breadth (B_A_ = 0.334) compared to yellow males (B_A_ = 0.265). In turn, a high value of food niche overlap between morphs was recorded (O_jk_ = 0.957). We found no differences between groups in the mean mass of stomach contents (red males: $\bar {X}$ = 0.76 ± 0.35 g; yellow males: $\bar {X}$ = 0.75 ± 0.27 g; *U*_24,21_ = 245, *P* = 0.89), stomach content volume (red males: $\bar {X}$ = 4159.7 ± 3605.6 mm^3^; yellow males: $\bar {X}$ = 3771.7 ± 3089.5 mm^3^; *t*_24,21_ = 0.23, *P* = 0.81), or the number of individual prey found in stomachs (red males: $\bar {X}$ = 13.8 ± 8.2 prey; yellow males: $\bar {X}$ = 11.7 ± 6.7 prey; *t*_24,21_ = 0.67, *P* = 0.50). Similarly, MANOVA on the full sample of males found no differences between morphs in either the volume and mass of stomach contents, or abundance of prey consumed between morphs (Wilks’ lambda = 0.98, *F*_(3,37)_ = 0.21, *P* = 0.88); likewise, there was no difference between morphs in these characteristics on a seasonal basis (Wilks’ lambda = 0.90, *F*_(6,74)_ = 0.66, *P* = 0.67). However, these characteristics varied across seasons independent of morph (Wilks’ lambda = 0.29, *F*_(6,76)_ = 10.49, *P* <0.01; mass: *F*_(2)_ = 5.41, *P* <0.01; volume: *F*_(2)_ = 28.3, *P* <0.001; abundance: *F*_(2)_ = 4.22, *P* = 0.02; Table 6).

**Table 6:** Descriptive statistics of response variables used in MANOVA of lizard stomach contents. For all measurements, the mean ± standard deviation is reported.

	Red			Yellow		
	Summer	Fall	Spring	Summer	Fall	Spring
Mass (g)	0.95 ± 0.43	0.55 ± 0.13	0.66 ± 0.20	0.80 ± 0.16	0.50 ± 0.21	0.85 ± 0.34
Volume (mm^3^)	7,097 ± 3,443	1,505 ± 543	1,873 ± 778	6,472 ± 2,950	1,239 ± 502	2,109 ± 731
Abundance	17.72 ± 7.34	9.4 ± 7.9	11.7 ± 7.8	14. 77 ± 8.74	10.8 ± 2.38	8.28 ± 4.11

**Notes.**

Mass mass of stomach contents volume volume of stomach contents abundance abundance of prey consumed

Correlation analyses found no significant relationship in either morph between SVL and volume of stomach contents (red males: *r* = 0.17, *P* = 0.42; yellow males: *r* = 0.25, *P* = 0.25), or between SVL and the total number of prey items found in stomachs (red males: *r* =  − 0.35, *P* = 0.09; yellow males: *r* = 0.025, *P* = 0.91). No relationship was found between total prey volume and either JL (*r*_s_ = 0.18, *P* = 0.29) or JW (*r*_s_ = 0.008, *P* = 0.96) in red males. However, there was a significant negative relationship between total prey volume and both JL (*r*_s_ = -0.44, *P* = 0.05) and JW (*r*_s_ = −0.45, *P* = 0.04) in yellow males.

## Discussion

We found no differences between morphs in any of the morphometric characters analyzed (Table 1). However, all morphs did exhibit allometric growth for all these same variables (Table 2), and showed seasonal allometric growth in the morphometric head traits (Table 3). These similarities may be a consequence of the size at which offspring are born, their rate of growth, and/or their body size at sexual maturity (Benabib, 2009). These similarities may also be a consequence of the homogeneity and availability of resources in the environment; that is, if resources (e.g., space, food) are sufficiently available, competition should be minimal or non-existent. In this context, morphological characters might not change; this is in contrast to a population of *S. minor* from La Manzana, Hidalgo (Stephenson, 2010), where morphometric differences were recorded among the morphs. At La Manzana, blue and red morph males were larger and exhibited proportionately larger heads than yellow morph males (Stephenson, 2010). Territorial behavior is common in heterogeneous environments where resources are grouped in space, for example, in the pine-oak forests of La Manzana with their intermittent limestone outcroppings that are important for *S. minor* at that locality (see Stephenson, 2010). The unequal distribution of resources can result in intense competition, with larger or more aggressive individuals at an advantage (Sinervo & Lively, 1996; Huyghe et al., 2007). Smaller or less aggressive individuals should adopt alternative strategies to obtain their resources (Gross, 1996; Sinervo & Lively, 1996), and such tactics may help explain the morphological, physiological, and behavioral differences seen among morphs in the La Manzana population (Stephenson, 2010). At El Enzuelado, the morphometric characteristics of the different color morphs are much more similar than at La Manzana; however, the aggressive and territorial behavior of the morphs at El Enzuelado may differ (McEvoy et al., 2013). Such behavior could result in an asymmetry in the retention of resources between morphs, so that the less aggressive or less dominant morph would have to use different tactics to obtain these resources (Sinervo & Lively, 1996), or move to environments where there is less competition (Paterson & Blouin-Demers, 2018). However, this interpretation should be treated with caution, since more detailed morphological and behavioral studies among the morphs of El Enzuelado and other populations of *S. minor*, and the distribution of resources in each environment, will be necessary in order to generate more robust conclusions.

We found that male *S. minor* lizards have an omnivorous diet, with 13 dietary categories represented. The diet category with the largest proportion in male stomachs was insects, followed by plant material (leaves and flowers), and arachnids (spiders and pseudoscorpions; Fig. 2, Tables 4 and 5). A high proportion of insects in the diet has been recorded in other studies of *S. minor* from both the same and different localities (García-Rosales et al., 2019), as well as in several closely related species [*S. torquatus* Wiegmann, 1828 (Feria-Ortiz, Nieto-Montes de Oca & Salgado-Ugarte, 2001); *S. jarrovii* Cope, 1875 (Gadsden et al., 2011)]. Consumption of insects is common in the diet of lizards in general, as these prey provide an excellent source of nutrients for growth, development, and daily activities (Gadsden et al., 2011; Zamora-Abrego & Ortega-León, 2016). The observed frequency of consumption of plant material was also high, confirming previous reports of this behavior in *S. minor* (García-Rosales et al., 2019), close relatives such as *S. mucronatus* Cope, 1885 (Méndez-De la Cruz, Casas-Andreu & Villagrán-Santa Cruz, 1992), and *S. torquatus* (Feria-Ortiz, Nieto-Montes de Oca & Salgado-Ugarte, 2001), and species of the lacertid genus *Podarcis* (Pérez-Mellado & Corti, 1993), among others. Potential benefits to consumption of plant material include increasing water uptake in dry environments (Méndez-De la Cruz, Casas-Andreu & Villagrán-Santa Cruz, 1992; Sazima, Sazima & Sazima, 2005; Serrano-Cardozo, Lemos-Espinal & Smith, 2008), supplementing nutritional intake when insect abundance is low (Greene, 1982; Búrquez, Flores-Villela & Hernández, 1986), improving the digestive process (Búrquez, Flores-Villela & Hernández, 1986), and reducing foraging time, given that plant material often offers a relatively abundant and accessible source of energy (Durtsche, 1992).

When examining diet at the level of color morph, we found that the diet of red males was composed of 12 diet categories, whereas 10 categories of prey were consumed by yellow males (Fig. 2, Table 4). The categories of diet not shared between morphs (e.g., Isoptera, Psocoptera) were those consumed by their respective morph in very low proportions. The number of prey categories consumed by each morph was different between morphs and seasons (Table 5). Despite this difference, the categories of diet with the highest values of food importance for both groups were Coleoptera, Orthoptera, and plant material (leaves and flowers). Notably, García-Rosales et al. (2019) found that the diet types consumed the most throughout the year by males and females of *S. minor* of this same population (but in years different from this study) were also Coleoptera, Orthoptera, and plant matter. The overrepresentation of these three groups in the diet of both morphs suggests that these categories are either especially abundant in the habitat of each morph, efficiently meet the nutritional requirements of all adult male lizards, or both. A similar diet composition among morphs corresponded to low niche breadth values for each morph, but a high niche overlap. The values of trophic niche breadth in both morphs are low because, of the total categories of prey that each morph consumes (red = 12 and yellow = 10), each is most frequently consuming only three main types of prey. On the other hand, the values of trophic niche overlap are high because both morphs share nine prey categories, and the prey that they do not share (four prey categories) are consumed in low proportions. These results indicate that males of *S. minor* have a specialist diet, because although food availability in the environment was not assessed, our data show a bias in consumption towards only a few categories of prey.

In addition, red and yellow morph diets were similar in terms of the amount of food ingested, as no significant differences were found between morphs in the weight or volume of stomach contents, or the number of individual prey found in stomachs. Similarly, there were no differences in these variables when analyzed by season. Similarities in diet might also reflect similar preferences for certain types of prey and/or tendencies to forage at similar sites at the same times of the day, as has been seen in other species of lizards (Wikelski & Trillmich, 1994). The observed similarity in the type and quantity of prey consumed by red and yellow males could also reflect similarity in head morphologies, since no differences were found between morphs in terms of traits linked to head size. However, the results obtained between seasons (summer, fall, and spring) should be taken with caution, since in some groups of data, the sample size was relatively small. On the other hand, across all males there were significant differences in the volume and mass of stomach contents and abundance of prey consumed across seasons of the year (Table 6). In general, a lower volume and weight of stomach contents was found in, and a lower abundance of prey was consumed by, both red and yellow morphs during the fall as compared to other seasons (though prey abundance for yellow morphs in the fall was higher than in the spring; see Table 6). In contrast, the largest volume and mass of stomach contents, as well as greatest abundance of prey consumed, were recorded in the summer (except for mass in the yellow morph, which was slightly higher in the spring; Table 6). Overall, the lowest values recorded for these variables in this population coincided with the period of courtship and mating, while the highest values were recorded in the pre-breeding season (Ramírez-Bautista et al., 2014). During reproductive activity, males ingest a smaller amount of food, since their activities during this time (especially courtship, mating, and territory defense) limit the time of foraging. Therefore, food intake is maximal during the pre-reproductive period in the spring and summer, with energy stored in fat bodies; this stored energy is utilized during the reproductive period, allowing males to decrease foraging and maximize their reproductive activities (Méndez-De la Cruz, Casas-Andreu & Villagrán-Santa Cruz, 1992; Ramírez-Bautista & Olvera-Becerill, 2004; Hernández-Salinas et al., 2010).

Correlation analyses showed that there was no significant relationship between SVL and volume of stomach contents, or between SVL and total number of prey items found in stomachs, in either morph. Previous work on this same species at El Enzuelado and La Manzana showed a relationship between SVL and volume of stomach content, but only when the analysis was performed at the population level with a comparatively large sample size (see García-Rosales et al., 2019); when the authors performed a similar analysis by sex for each population, no significant correlations were observed (García-Rosales et al., 2019). The lack of significant correlations between SVL and stomach contents of males in this study might therefore reflect limited sample size, rather than the absence of such patterns in general (García-Rosales et al., 2019). Future studies should therefore consider performing these analyses with a larger sample size, if feasible, to account for this possibility. Indeed, other studies with polymorphic species have revealed morphometric differences between morphs in body structures, particularly in head anatomy (Huyghe et al., 2007; Bustos-Zagal et al., 2014). Such differences in head size may be linked to variation in bite force, which in turn may be associated with morph differences in diet on the basis of prey size (Gadsden et al., 2011; Ngo et al., 2015) or hardness (Huyghe et al., 2007). We also found no relationship between the total volume of prey and either JL or JW in red males, but significant and negative associations between these variables were observed for yellow males (JL: *r*_s_ = −0.44, *P* = 0.05; JW: *r*_s_ = −0.45, *P* = 0.04). Contrary to our expectations, yellow males with larger jaws consumed smaller prey; however, this result should be taken with caution, since prey volume data only included complete prey items (i.e., those that were not crushed) and hence excluded those prey that were not fully intact. In addition, at least some non-intact prey items were found in the stomachs of all examined individuals. Given that large prey must be crushed for swallowing to be feasible, whereas small prey consumed by a lizard with large jaws could potentially be swallowed whole, large intact prey—i.e., those closest to the limits imposed by lizard jaw dimensions—are likely underestimated in this analysis as compared to small prey, and hence their influence on the relationship of jaw size to prey volume is disproportionately reduced. Consequently, future studies will need to implement a different approach that more reliably assesses the relationship between mandibular dimensions and prey volume.

Morph-specific differences in diet have been reported between morphs in some other lizard species that show polymorphism (Lattanzio & Miles, 2016; Scali et al., 2016). The lack of such a pattern in *S. minor* from El Enzuelado could be a function of environmental homogeneity at the study site, such that food resources may be relatively uniform throughout the area. Similarly, it has been observed that both morphs exploit the same types of microhabitat (boulders and rock piles; A. García-Rosales, pers. obs., 2017), such that both groups of males may be exposed to the same type and amount of food. In this regard, Lattanzio & Miles (2016) noted that segregation of the microhabitat that arises from behavioral interactions between males of polymorphic species exposes individuals of each morph to different types of resources, resulting in a trophic polymorphism in species that exploit ARTs. In this study, the similarities in diet between morphs suggest that either the behavior of the morphs is similar, morphs exploit the same microhabitats, or both.

On the other hand, Scali et al. (2016) proposed the hypothesis that each morph in a polymorphic species has evolved a specific preference for certain types of prey, only partially shared with another morph. This segregation of trophic niches would reduce competition for resources, thus promoting coexistence of morphs in the same locality (Pianka, 1974). In this study, we found no differences in the diet of the analyzed morphs. This suggests that male *S. minor* from El Enzuelado live in an environment with relatively abundant food resources, such that any competition between morphs may be minimal, or that the polymorphism in this population is probably not maintained by niche partitioning.

## Conclusions

In conclusion, our results showed that there are no morphological differences among morphs of *S. minor* from El Enzuelado for any morphometric trait examined, in contrast to that reported for populations elsewhere (Stephenson, 2010). In addition, we found no differences in the diet of red and yellow morphs, which were very similar in terms of the type and quantity of ingested food, rejecting the hypothesis that morphs in this population are maintained by niche partitioning. However, future studies of this species should include dietary analyses of different populations that exhibit heterogeneity in the structure of the habitats used by males. Such studies should also monitor patterns of diet over a longer period of time, since territorial behavior, and consequently spatial segregation, may change according to environmental conditions (Knapp et al., 2003). Researchers should also continue studies on the behavior of males in this species testing for morph-specific differences in aggressive behavior (Stephenson, 2010). Such studies may collectively provide insights into the basis for population variation in ARTs in polymorphic species.

##  Supplemental Information

10.7717/peerj.8099/supp-1 Supplemental Information 1 Data base Morphology and diet in S. minor

## Additional Information and Declarations

### Field Study Permissions

The following information was supplied relating to field study approvals (i.e., approving body and any reference numbers): All animal use was approved under collecting permit SGPA/DGVS/06183/17 issued by the Secretaría del Medio Ambiente y Recursos Naturales (SEMARNAT) of the Government of Mexico.

### Data Availability

The following information was supplied regarding data availability: Raw data is available as a Supplemental File.
